# Benefit of Bovine Viral Diarrhoea (BVD) Eradication in Cattle on Pestivirus Seroprevalence in Sheep

**DOI:** 10.3389/fvets.2021.681559

**Published:** 2021-10-04

**Authors:** Andrea Franziska Huser, Jessica Grace Schär, Claudia Bachofen, Elena de Martin, Jasmine Portmann, Hanspeter Stalder, Matthias Schweizer

**Affiliations:** ^1^Institute of Virology and Immunology, Bern, Switzerland; ^2^Institute of Virology, Vetsuisse Faculty, University of Zurich, Zurich, Switzerland; ^3^Department of Infectious Diseases and Pathobiology, Vetsuisse Faculty, University of Bern, Bern, Switzerland; ^4^Graduate School for Cellular and Biomedical Sciences, University of Bern, Bern, Switzerland

**Keywords:** pestivirus, bovine viral diarrhoea virus (BVDV), border disease virus (BDV), persistent infection, seroprevalence, virus transmission, Switzerland, eradication

## Abstract

Bovine viral diarrhoea virus (BVDV) and Border disease virus (BDV) are closely related pestiviruses of cattle and sheep, respectively. Both viruses may be transmitted between either species, but control programs are restricted to BVDV in cattle. In 2008, a program to eradicate bovine viral diarrhoea (BVD) in cattle was started in Switzerland. As vaccination is prohibited, the cattle population is now widely naïve to pestivirus infections. In a recent study, we determined that nearly 10% of cattle are positive for antibodies to BDV. Here, we show that despite this regular transmission of BDV from small ruminants to cattle, we could only identify 25 cattle that were persistently infected with BDV during the last 12 years of the eradication program. In addition, by determining the BVDV and BDV seroprevalence in sheep in Central Switzerland before and after the start of the eradication, we provide evidence that BVDV is transmitted from cattle to sheep, and that the BVDV seroprevalence in sheep significantly decreased after its eradication in cattle. While BDV remains endemic in sheep, the population thus profited at least partially from BVD eradication in cattle. Importantly, on a national level, BVD eradication does not appear to be generally derailed by the presence of pestiviruses in sheep. However, with every single virus-positive cow, it is necessary to consider small ruminants as a potential source of infection, resulting in costly but essential investigations in the final stages of the eradication program.

## Introduction

Bovine viral diarrhoea virus (BVDV) and Border disease virus (BDV) are closely related pestiviruses. BVDV is an important cattle pathogen with a worldwide distribution, and due to its economic impact, eradication programs are ongoing in several European countries (1). The closely related BDV is a pathogen of sheep and has been isolated from this species in all continents where sheep are reared (2). However, in contrast to BVDV, there are no known attempts to control or eradicate this virus. A common feature of the two viruses is their ability to persistently infect the foetus when the dam or ewe is infected early in gestation. While the pregnant animals usually show no or only mild clinical signs, develop neutralising antibodies, clear the virus and are immune to re-infection, the foetus accepts the virus as “self” and becomes immunotolerant to the infecting virus strain. Hence, the foetus may develop normally and remains persistently infected (PI) for life. Such animals constantly shed large amounts of virus, representing the most important source of infection for naïve animals and are crucial for the persistence of BVDV in the host population (3). PI animals may be free of clinical signs; more often, however, they show growth retardation and have a reduced life expectancy (4–7). While BDV PI lambs may show the pathognomonic signs of rhythmic tremor, ataxia, and an abnormal, hairy fleece (hence referred to as “hairy shakers”), the clinical signs in BVDV PI calves are usually less specific and may range from recurrent diarrhoea to pneumonia (8, 9). With increasing age, PI calves may develop mucosal disease. This lethal manifestation of the BVDV infection is associated with a change of the virus from non-cytopathogenic to cytopathogenic and is characterised by mucosal erosions and untreatable diarrhoea (10).

Pestiviruses are not strictly species specific. Especially BVDV is known to infect a wide range of domestic and wild even-toed ungulates. Sheep PI with BVDV have been reported frequently, both as the result of experimental or natural infections of pregnant ewes (11–14). By contrast, interspecies transmission of BDV seems to be rare (8). Due to the genetic and antigenic relatedness of pestiviruses, most routine diagnostic tools used for detection of BVDV cross-react with BDV strains, which impedes routine differentiation of these ruminant pestiviruses (8, 15).

The finding of cattle that are PI with BDV is of concern mainly in countries that have ongoing BVD eradication programs, as it exacerbates contact tracing and identification of the source of infection. In 2008, Switzerland started a mandatory national BVD eradication program in cattle (15). During the first year, all cattle were tested for antigen or viral RNA and animals identified as PI were eliminated. In the following years, all newborn calves were similarly screened and in 2013, surveillance was switched to testing for pestivirus-specific antibodies, either in the blood of young calves (“spot test”), in milk of first-lactating cows, or in bulk milk (15–19). Prior to the start of the eradication program, around 1.3% of all newborn calves and 0.7% of all cattle were PI, and ~60% of the cattle population was seropositive (17, 20). None of the herds in Switzerland were devoid of seropositive animals (20) despite vaccination being very uncommon at that time. Over the course of the eradication program, the epidemiological situation changed markedly. Whilst PI animals were detected in some 12% of herds before the start of the program (20), currently ~99.5% of all cattle herds are certified free of BVDV (15). Since the start of the eradication, vaccination was prohibited in Switzerland as this would interfere with serology as a tool to monitor the progress of the program. However, monitoring the effect of any BVD eradication program on the epidemiological status is not without pitfalls. First, the control program involves only BVDV, and not BDV. Second, since BVDV is not strictly restricted to cattle and BDV not to small ruminants, transfer of pestiviruses back from uncontrolled host species to cattle must be avoided, especially as the sinking seroprevalence in cattle might facilitate interspecies transmission. In fact, naturally occurring cattle PI with BDV have been reported in various countries in Europe [including Switzerland (19, 21)] and elsewhere, e.g., in New Zealand [for review, see (8)]. Moreover, we observed that ~7% of all pestivirus antibody-positive cattle sera collected in Switzerland between 2012 and 2014, i.e., four to six years after the start of the BVD eradication program, were reactive to BDV. Accordingly, keeping small ruminants, especially sheep, together with cattle was identified as the highest risk factor for harbouring BDV seropositive cattle (22).

Here, we describe the discovery of BDV PI cattle in Switzerland that were detected in the first decade of the BVD eradication program. As (i) the majority of these cattle were detected in Central Switzerland, and (ii) the highest BDV-seroprevalence in cattle was found in the same area (22), we investigated the impact of BVD eradication in cattle on the BVDV- and BDV-seroprevalence in sheep in Central Switzerland. With these data, a clearer picture of the role of sheep as a virus reservoir for BDV as well as BVDV might be drawn, knowledge that is essential to reduce the economic burden of BVD eradication programs, especially in the final stage. In addition, it will provide evidence whether the sheep population benefits from eliminating BVDV in cattle.

## Materials and Methods

### Identification of BDV Persistently Infected Cattle

Cattle persistently infected with BDV were detected within the framework of the Swiss BVD eradication scheme as described (17, 21). Animals were initially tested for the presence of pestivirus antigen by ELISA or pestiviral RNA by real-time RT-PCR. This initial test was performed by designated regional diagnostic labs using commercial kits approved for the BVD eradication in Switzerland. To confirm positive results, EDTA blood samples were taken ~2 weeks after the initial test and sent to the Institute of Virology and Immunology, the national reference laboratory for pestiviruses. If routine diagnostic quantitative RT-PCR confirmed the presence of a pestivirus, samples were directly sequenced in the 5'-untranslated region (UTR) in order to determine the pestivirus species, genotype and subgroup (21).

### Samples for Serology

Samples used in this study were collected in Switzerland in 2001 and in 2016–2017, i.e., 7 years prior to and ~8–9 years after the start of the Swiss BVD eradication program in cattle. Sera from the year 2001 were available at our institute and were originally collected for a study on sheep scab and are mainly from Central Switzerland with canton Schwyz as the core area. In the years 2016–2017, samples of anticoagulated (EDTA) blood were collected within the scope of brucella surveillance in Switzerland, and samples from Central Switzerland were transferred to our laboratory thereafter. Thus, a total of 1,247 sheep samples from 133 farms (2–20 samples collected per farm; average 9.4, median 9.0) collected in 2001 and 1,584 samples from 83 farms (1–50 samples collected per farm; average 18.9, median 14.5) obtained in 2016/17 were stored at −20°C and used for serology (Table 1). A sufficient number of samples for both time points were only available from the Canton Schwyz (SZ) (Table 1), and statistical analysis was therefore only performed with data from this Canton. Samples from the Canton SZ were randomly selected and originate from all over the Canton in both sampling time points, representing ~6 and 3% of all sheep in the Canton (data from the Federal Statistical Office) in 2001 and 2016/2017, respectively.

**Table 1:** Number, year of sampling and origin of sheep sera analysed.

**Year**	**Canton**	**Samples**	**Farms**	**Communities**
2001	SZ	1,121	131	53
	UR	26	2	2
	Total	1,247	133	55
2016/2017	LU	287	18	16
	NW	103	4	4
	OW	123	9	7
	SZ	617	29	19
	UR	361	18	13
	ZG	93	5	4
	Total	1,584	83	63

*LU, Lucerne; NW, Nidwalden; OW, Obwalden; SZ, Schwyz; UR, Uri; ZG, Zug*.

### ELISA

All samples were tested for antibodies to pestiviruses using an “in-house” ELISA (23, 24). This ELISA does not distinguish between BD- and BVD antibodies. As conjugate to detect antibodies from non-bovine species, protein-G-peroxidase (Thermo Fisher, recombinant protein G-peroxidase, diluted 1:2000) was used. The optical density (OD) of the chromogen ABTS was read at 405 nm and the value of the sample was expressed in percentage of the OD of the standard serum. Relative values above 30% were considered positive, whereas values below 20% were considered as negative. Values between 20 and 30% were considered inconclusive (25).

### Serum Cross Neutralisation Test

All samples positive in the antibody ELISA were further investigated by serum neutralisation test (SNT), the gold standard in serology and the method of choice to detect virus-specific antibodies (26). In order to differentiate the source of infection, i.e., BVDV or BDV, we performed cross-neutralisation tests using different strains of ruminant pestiviruses as challenge virus as described (22), except that the sera were initially 8-fold pre-diluted instead of 10-fold. Samples with BVDV and BDV SNT titers higher than 6 were regarded as positive. In this previous work, we determined that the use of two BVDV strains (BVDV-1a and BVDV-1h) and one BDV strain (BDswiss/BDV-8) provided the best discriminatory power to differentiate antibodies to BVDV and BDV. Differentiation was made by calculating the (reverse) quotient of antibody neutralisation titers of BVDV-1a/BDV and BVDV-1h/BDV (larger value in the numerator, with a value of 6 being used for negative samples to formally calculate a ratio). Ratios >4 were considered to be significant and assigned to BVDV or BDV, whereas ratios below 4 were described as indeterminate (22, 27). The final assignment of a sample was done as described (22).

### Detection of Viral RNA in Sheep Samples

All sheep samples that were classified as antibody negative or indeterminate in this study (i.e., all samples that were either negative in the antibody ELISA or which, in the SNT, were rated negative or could not be classified based on their toxic effect on cell cultures) were tested for the presence of pestiviral RNA by RT-PCR as described (19, 28) with minor modifications: RNA extraction was done on a KingFisher Flex System (Thermo Fisher Scientific, Reinach, Switzerland) using the NucleoMag VET Kit (Macherey-Nagel, Oensingen, Switzerland) according to the manufacturers' protocol, and RT-PCR was performed following the protocol by QuantiTect^®^ Probe RT-PCR Kit (Qiagen AG, Hombrechtikon, Switzerland) using an ABI 7300 Real-Time PCR instrument and software package (Applied Biosystems, Foster City, CA USA). To monitor the efficiency of the RNA isolation of each sample, Sendai virus was added representing a control RNA protected within the virus particle from RNase degradation. A defined amount yielding a final Ct value of ~25 was added to each sample prior to RNA isolation. Thus, the performance of the RNA isolation and the RT-PCR reaction could be evaluated for every individual sample (29).

Negative results were labelled with the maximal number of cycles performed, i.e., 45, whereas samples with Ct < 31 were rated as positive and with Ct ≥ 31 as weak-positive. Samples were tested in pools of 10 and pools yielding a weak-positive result were repeated. Samples from positive or twice weak-positive pools were subsequently tested individually.

### Statistics

The data collected before the start of the eradication program in 2001 were compared with the data collected in 2016–2017 using the statistic software RStudio (RStudio: Integrated Development for R. RStudio, PBC, Boston, MA). To measure the precision of the sampled population as representative for the whole population, the standard error SE of each proportion has been calculated and used to derive the 95% confidence interval for each population. The proportions of the two independent population groups were then compared using the Chi-squared test with the Yates' correction, considering as null hypothesis that the seroprevalence of BDV in the sheep population is not influenced by the BVD eradication program. *P* values lower than 0.05 were considered as significant.

## Results

### Cattle Persistently Infected With BDV

**Figure 1:**
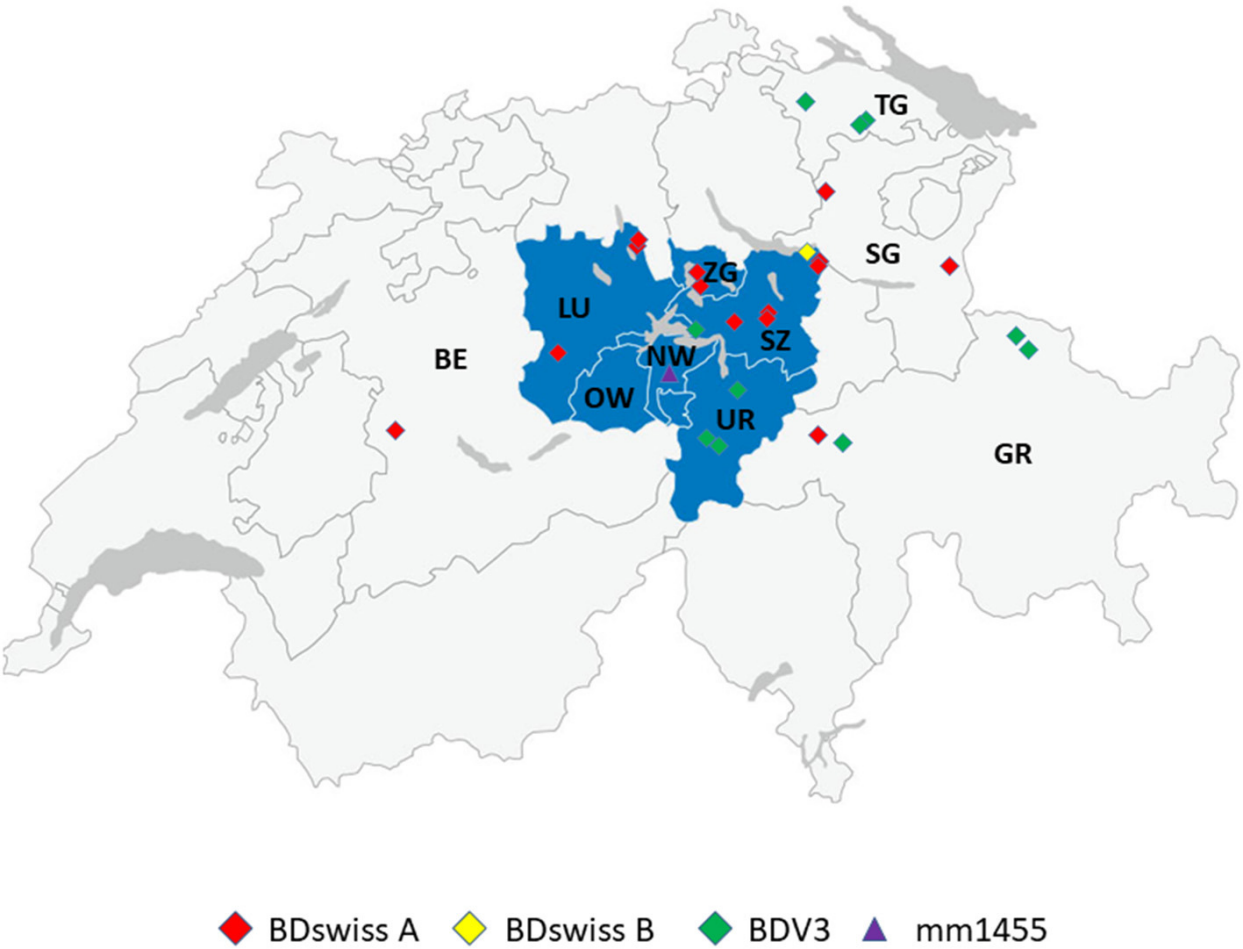
Geographic distribution of the place of birth of cattle persistently infected with BDV with the subgenotype BD-3 in green, BDswiss-A in red and BDswiss-B in yellow. The location of the farm where the persistently infected sheep (mm1455) was identified is shown in purple. The corresponding cantons were labelled as Bern (BE), Luzern (LU), Nidwalden (NW), Obwalden (OW), Uri (UR), Schwyz (SZ), Zug (ZG), St. Gallen (SG), Graubünden (GR), and Thurgau (TG). The cantons of Central Switzerland are stained in blue.

In the context of BVD eradication in Switzerland, routine sequencing (19) of 9'493 BVDV positive samples taken from 2008 through 2020 revealed that 25 supposedly BVDV-positive calves were infected with BDV instead (Table 2). All but one cases originated from the Central and Eastern regions of Switzerland, namely the cantons Schwyz (SZ), Luzern (LU), Graubünden (GR), Uri (UR), St. Gallen (SG), Thurgau (TG) and Zug (ZG), with the majority (60%) of the cases being located in Central Switzerland and 36% in Eastern Switzerland (Figure 1). The majority of the cases were detected in the years 2008–2012 and 2019–2020. In 80% of the cases, the cattle had contact to sheep (Table 2, column “Sheep contact”), either on the same or the neighbouring farm, as revealed by farm visits or based on information from the national animal movement database (18). In six cases where contact to sheep was reported, we were able to determine the seroprevalence of pestivirus antibodies in these sheep flocks (Table 2, column “Sheep seroprevalence”). In five cases the seroprevalence was remarkably high (62 to 90%), while in one case it was only 16%. In the latter case, we also tested the (larger) sheep flock of the neighbouring farm and found a seroprevalence in this flock of 53% (data not shown). However, no viremic sheep were detected in these two flocks. Pestivirus infected sheep were present in three out of the 7 flocks tested (Table 2, where indicated in the column “Sheep contact”).

**Table 2:** Cattle persistently infected with BDV identified in Switzerland since the start of the BVD eradication in 2008 until the end of 2020.

**Sample**	**Year**	**Genotype**	**Canton**	**Sheep contact**	**Sheep seroprevalence**	**GenBank**
boBD-CH5	2008	BD3	SZ	Yes, PI sheep	69% (*n* = 52)	MH908082
boBD-CH2	2009	BD3	GR	Yes	n.i.	MH908079
boBD-CH1	2009	BDswiss	SG	Yes, PI sheep	89% (*n* = 72)	MH908078
boBD-CH4	2010	BDswiss	SZ	Yes	n.i.	MH90808
boBD-CH3	2010	BD3	UR	Yes (no PI)	62% (*n* = 74)	MH908080
boBD-CH9	2011	BDswiss	GR	Yes (no PI)	16% (*n* = 68)	MH908084
R9336/11	2011	BDswiss	SZ	Yes (no PI)	70% (*n* = 20)	MF102261
boBD-CH8	2011	BDswiss	ZG	Yes	n.i.	MH908083
boBD-CH10	2012	BDswiss	SZ	Yes (no PI)	88% (*n* = 8)	MH908085
boBD-CH11a	2012	BDswiss	SZ	Yes	n.i.	MH908086
boBD-CH11b	2012	BDswiss	SZ	Yes	n.i.	MH908087
boBD-CH12^#^	2012	BDswiss	SZ	Yes, 2 PI sheep	n.i.	MH908088
boBD-CH13a	2012	BDswiss	LU	Yes	n.i.	MH908089
boBD-CH13b	2013	BDswiss	LU	Yes	n.i.	MH908090
boBD-CH14	2015	BDswiss	ZG	Yes	n.i.	MH908091
boBD-CH15	2015	BD3	GR	Yes	n.i.	MH908092
boBD-CH16	2016	BD3	TG	Yes	n.i.	MH908093
boBD-CH17	2019	BDswiss	BE	Yes	n.i.	MW659875 ^*^
boBD-CH18	2019	BDswiss	LU	Yes	n.i.	MW659876 ^*^
boBD-CH19	2019	BD3	UR	No	n.i.	MW659877 ^*^
boBD-CH20	2019	BD3	UR	No	n.i.	MW659878 ^*^
boBD-CH21	2020	BD3	GR	Yes	n.i.	MW659879 ^*^
boBD-CH22	2020	BDswiss	SG	No (goats)	n.i.	MW659880 ^*^
boBD-CH23	2020	BD3	TG	No	n.i.	MW659881 ^*^
boBD-CH24	2020	BD3	TG	No	n.i.	MW659882 ^*^

*Sample identification, year of sampling, (sub-) genotype of BD virus strain identified, origin (canton) of the affected cattle, and, where available, further information to the case, i.e., whether possible contacts to sheep were reported, whether a PI sheep or not was detected (stated if investigated), and the seroprevalence of the sheep herd in contact, are indicated. AG, Aargau; BE, Bern; GR, Grisons; LU, Lucerne; SG, St. Gallen; SZ, Schwyz; TG, Thurgau; UR, Uri; ZG, Zug; n.i.: not investigated*.

# *Might have been transiently infected*.

* *This study*.

### Pestivirus Seroprevalence in Sheep

From the samples collected in 2001, 267 out of 1,247 tested positive for the presence of antibodies to pestiviruses, yielding a seroprevalence of 22.0% (Table 3). From those taken in 2016/2017, 282 out of 1,584 tested positive, accounting for an overall seroprevalence of 18.4% (Table 4). In both groups of samples, 2.5–3% gave an inconclusive result and were excluded from calculating the seroprevalence. Due to limited amount of sample material available, the ELISA tests of the samples with an inconclusive result were not repeated. A small number of samples positive in ELISA turned out to be false positive, as they were classified as negative in SNT (see next chapter). This difference probably originates from the higher specificity of the neutralisation test and the fact that the ELISA largely detects antibodies to the non-structural protein NS2-3, whereas the structural protein E2 represents the main target of neutralising antibodies (30). Subtracting these negative samples from the ELISA results, however, does not substantially affect the interpretation of the results (Supplementary Tables 1, 2). Approximately half of the farms contained antibody-positive animals at both time points (61 and 47% in 2001 and 2016/2017, respectively).

**Table 3:** Pestivirus seroprevalence and number of farms harbouring seropositive animals (“farms affected”) per canton according to the ELISA results of sheep sera collected in 2001.

**Canton**	**Positive**	**Negative**	**Inconclusive**	**Seroprevalence**	**Farms affected**
SZ	262	928	31	22.0%	79 (60%)
UR	5	21	0	19.2%	2 (100%)
Total	267	949	31	22.0%	81 (61%)

*Samples with inconclusive ELISA results were omitted from the calculation of the seroprevalence*.

**Table 4:** Pestivirus seroprevalence and number of farms harbouring seropositive animals (“farms affected”) according to the ELISA results of sheep sera collected in 2016–2017.

**Canton**	**Positive**	**Negative**	**Inconclusive**	**Seroprevalence**	**Farms affected**
LU	10	272	5	3.5%	4 (22%)
NW	42	60	1	41.2%	4 (100%)
OW	39	83	1	32.0%	3 (33%)
SZ	86	504	27	14.6%	16 (55%)
UR	68	279	14	19.6%	10 (56%)
ZG	37	56	0	39.8%	2 (40%)
Total	282	1,254	48	18.4%	39 (47%)

*Samples with inconclusive ELISA results were omitted from the calculation of the seroprevalence*.

Most samples (97.9%) taken prior to the start of the BVD eradication program originated from the canton of Schwyz (SZ). By contrast, samples collected in 2016/2017 were obtained from all the cantons of Central Switzerland (Table 1; Figure 1). The seroprevalence in the individual cantons varied from 3.5 to 41.2%, with 22–100% of the farms being affected (Tables 3, 4). The overall seroprevalence on the animal level was slightly higher in 2001 compared to ~8 years after the start of the eradication in cattle. Nevertheless, statistical evaluation was performed only with the data obtained from the canton of SZ, where samples from both time points were available. The proportion of seropositive animals and the corresponding 95% confidence interval were 0.22 (95% CI: 0.19, 0.25) and 0.146 (95% CI: 0.11, 0.17) in 2001 and 2016/2017, respectively (Tables 3, 4). Using a Chi-squared test, we evaluated whether the discrepancy between the expected and the calculated frequencies of ELISA positive animals in 2001 and 2016/2017 was sufficient to reject the null hypothesis of having no significant difference between both time points. The discrepancy was significant [X^2^ (1, *N* = 1780) =13.48, *P* < 0.05]. These results indicate that the pestivirus seroprevalence is significantly reduced after compared to prior to the start of BVD eradication, with a prevalence ratio (PR) between the ELISA-positive sheep in 2001 and 2017 of 0.66 (95% CI: 0.52, 0.82; *P* < 0.001). Therefore, the animals were 0.66 times as likely to be ELISA-positive after the eradication program compared to the animals before the start of the eradication program. This significant difference in the PR is maintained [0.71 (95% CI: 0.56, 0.90, *P* < 0.01)] when excluding the samples that tested negative in SNT (see below), i.e., that were false positive in ELISA (Supplementary Tables 1, 2).

### Differentiation of Antibodies in Sheep Sera by Cross-SNT

All sheep sera that were seropositive by ELISA were tested by cross-neutralisation to differentiate between a humoral immune response to BVDV and BDV. Due to the rather long duration of storage, the samples from 2001 were generally of lower quality than those from the later time point, exemplified by 54 of these sera being toxic to cell cultures, compared to only 13 from 2016/2017 (Supplementary Tables 3, 4).

In 2001, 13.3 and 60.7% were assigned to contain neutralising antibodies to BVDV and BDV, respectively. By contrast, 1.5 and 90% of the samples from 2016/2017 were assigned to BVDV and BDV, respectively (Table 5). Using three different challenge viruses for the cross-SNT (22), only 20 (in 2001) and two (in 2016/2017) samples remained “indeterminate” and thus, it was possible to assign 90.5 and 99.3% of all samples from 2001 and 2016/2017, respectively. Only one sample in each group provided contradictory results, i.e., an assignment to BVDV or BDV based on the two pairs of challenge viruses (Supplementary Tables 3, 4). Due to the lack of sufficient material, these SNTs could not be repeated and both samples were rated as indeterminate. All other samples rated as indeterminate had a BD/BVD quotient that did not exceed 4.

**Table 5:** Differentiation of antibodies in sheep sera collected in 2001 and 2016/2017 by cross-SNT.

**Year of sampling**	**ELISA-pos. sera [n]**	**Assignment**	**Proportion [n]**	**Proportion [%]**
2001	267	BVDV	28	13.3
		BDV	128	60.7
		Indeterminate	20	9.5
		Negative	35	16.6
		Total assigned	211	100
2016/2017	282	BVDV	4	1.5
		BDV	242	90.0
		Indeterminate	2	0.7
		Negative	21	7.8
		Total assigned	269	100

*ELISA-positive sera that could not be assigned were either toxic to cell cultures or unavailable. For more details to the assignment, see Supplementary Tables 3–6*.

Based on the differentiation of the antibodies to pestiviruses, it appears evident that the number of sheep harbouring antibodies to BVDV was strongly reduced during the BVDV eradication program in cattle. Using again only the data of the canton of SZ (Supplementary Tables 5, 6), the prevalence ratio (PR) of sheep being BVDV-positive prior to the start of eradication in cattle compared to the later time point was 4.219 (95% CI: 1.32, 13.49; *P* < 0.1), while a PR of 1.35 (95%: CI 1.19, 1.52; *P* < 0.0001) was observed for being BDV positive after compared to prior start of the BVD eradication programme.

### Detection of Viral RNA in Sheep Samples

**Figure 2:**
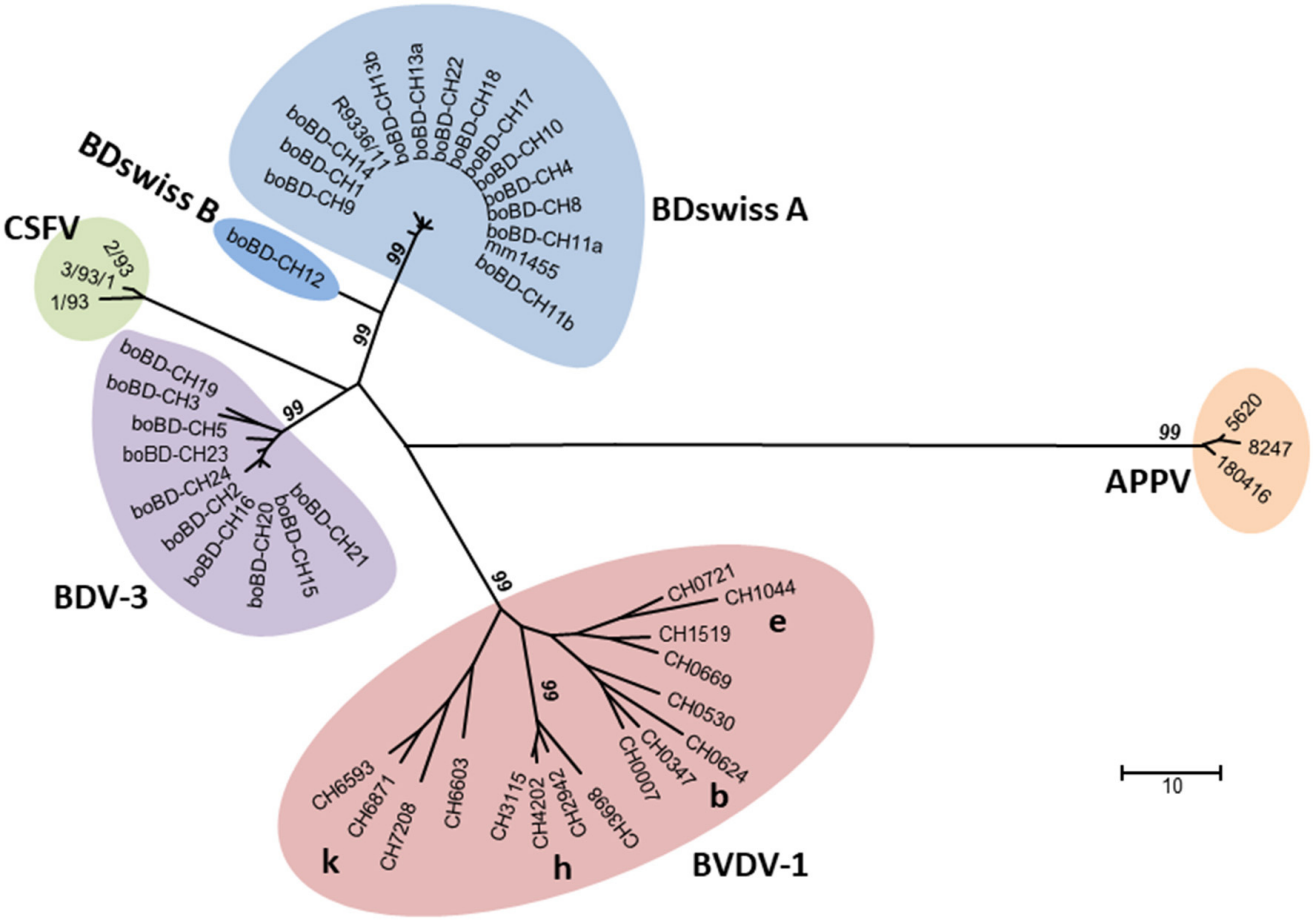
Phylogenetic analysis of nucleotide sequences encompassing the 5'-UTR of the pestiviral RNA genome. All the major sub-genotypes of pestiviruses described in Switzerland are included in the tree, i.e., BDV-3 and BDswiss (BDV-8) with samples from this study, representative samples of BVDV-1b, -1e, -1h, and -1k (21), strains of atypical porcine pestiviruses found in domestic pigs in Switzerland (29), and strains of classical swine fever virus isolated in Switzerland in the last outbreak in wild boars around the year 2000 (31). The evolutionary history was inferred using the neighbour-joining method. The genetic analysis was calculated, and the figure prepared as described in Supplementary Table 7. The numbers close to the branches represent the values (%) of 1,000 bootstrap replicates, and only values ≥99 are indicated. Line lengths are proportional to genetic distance and are in the units of the number of base differences per sequence, as indicated by the scale bar.

All sheep sera that were classified as pestivirus antibody negative or inconclusive (see Methods section) were analysed by RT-PCR for the presence of viral RNA. Due to insufficient sample volume, 19 sera (16 from 2001 and three from 2016/2017) could not be tested. After individual testing of samples from initially positive or twice weakly-positive pools, only one single sample (labelled as mm1455) turned out to be positive with a Ct value of 24.8. Sequencing part of the 5'-UTR revealed that the pestivirus belongs to the BDswiss (BDV-8) subgroup of ruminant pestiviruses (Figure 2). The sample was collected in 2017 in the canton of Nidwalden (NW; Figure 1). From this herd, 40 samples were analysed in this study, of which 33 (82.5%) were positive by ELISA. All seropositive samples from this farm except one that was toxic in cell culture could be assigned to BDV by cross-SNT, with SNT titers against BDV of 152–1,218 (average 647; median 609).

## Discussion

The economic impact of infection with BVDV on cattle farming has led to eradication programs in many countries (15, 32–36). Although it has been known for many years that BVDV may also infect sheep and that BDV, mostly found in sheep, may infect cattle, the possible implications for the pestivirus status in these two species are little studied (8, 22, 37, 38). On the one hand, BVDV and BDV are closely related, but on the other hand, both viruses are genetically and antigenically highly diverse within their own species, making specific diagnostics rather elaborate and expensive. In addition, it was unknown whether the absence of vaccination would make it easier for BDV to get a foothold in cattle. Similarly, decreased transfer of BVDV from cattle to sheep might alter the epidemiology of pestiviruses in sheep.

Here, we show that, despite serological evidence of regular transfer of BDV from sheep to cattle (22), we identified to date <30 cattle which were PI with BDV. This strongly indicates that the successful establishment of persistent infections in cattle upon cross-species infection by BDV from sheep does occur but is a rare event. By comparing the epidemiological situation in sheep before and after the start of the mandatory BVD eradication program in cattle, we provide strong evidence that pestiviruses remain endemic in the sheep population, but that the seroprevalence to BVDV strongly decreased after its eradication in cattle. Genotyping the BD viruses identified in sheep and cattle revealed that the same type of viruses could be found in both species, further indicating cross-species infection from sheep to cattle and vice versa.

### Infrequent Generation of Cattle Persistently Infected With BDV

Between the start of the BVD eradication in 2008 and the end of 2020, we identified only 24 calves by nucleotide sequencing that were persistently infected with BDV out of close to 10,000 samples sequenced. An additional case that was initially suspected to be persistently infected finally turned out as transiently infected (Table 2, boBD-CH12). This calf was positive by real-time RT-PCR for ~3 months but with high Ct values [compare Figure 3B in (15)]. Despite most BDV persistently infected cattle were identified in Central and Eastern Switzerland (Figure 1), there was no obvious correlation between the single cases. However, in the majority of cases, contact between sheep and cattle could have been possible, as observed on site or due to the presence of sheep on the farm according to the animal movement database (18). A sampling bias for this clustering can be excluded, as 46.7% of the samples sequenced during the eradication program were obtained from Western Switzerland. Similarly, the uneven distribution of BDV-infected cattle in Switzerland is not just based on the number of sheep present in a given area, as only around 15 and 50% of Swiss sheep are located in Central and Eastern Switzerland, respectively (data from the Federal Statistical Office). Rather, regional traditions of keeping cattle and sheep together might facilitate interspecies transmission, but data on the corresponding herd management practises are not available. However, it is corroborated by the facts that (i) the main risk factor for detecting BDV-specific antibodies in cattle was found to be the contact with small ruminants, mainly sheep (22), and (ii) that Central and Eastern Switzerland are similarly the main hot spots for malignant catarrhal fever (MCF). This mostly lethal disease is caused by transmission of ovine herpesvirus 2 (OvHV-2) from the ovine reservoir hosts to indicator hosts such as cattle. Close contact, particularly after lambing, is known to be a major risk factor for MCF (39). The OvHV-2 positivity rate of suspected MCF cases is significantly higher in Central and Eastern Switzerland compared to other regions that submit relevant numbers of samples for testing (personal communication by C. Bachofen; Swiss MCF reference laboratory).

### Pestivirus Seroprevalence in Sheep

The ELISA results showed a pestivirus seroprevalence in sheep of 22.0 and 18.4% in Central Switzerland prior to and 8 years after the start of BVD eradication, respectively. With 22.0 and 14.5%, the values for the canton SZ, where most of the samples in 2001 originate from, were in a similar range (Tables 3, 4). These values of the pestivirus prevalence observed in this study are in accordance with previous studies that reported values of 13.5–22% in sheep (40–43). This is considerably lower than the pestivirus seroprevalence in cattle, which was ~60% in Switzerland prior to eradication (20). Nevertheless, around half of all sheep farms was affected, i.e., owned antibody-positive sheep, which indicates that pestiviruses are widely circulating in the sheep population. Overall, the decrease in seroprevalence in sheep after the BVDV eradication in cattle, despite being significant for the Canton of Schwyz, is not pronounced and might have occurred by chance due to sampling variability, even though the farms sampled were well-distributed from all over the Canton.

### Differentiation of Antibodies to BDV and BVDV

Applying our recently optimised cross-neutralisation assay (22) to the sheep sera, we were able to determine the antibody specificity of >90% of all samples. This represents a clear improvement compared to the previously used cross-SNT using only one BVDV-1 and a single BDV strain, where 30 to 66% of cattle, sheep, or goat sera could not be assigned to one of the ruminant pestiviruses (22, 41, 44). The majority of antibodies were assigned to BDV (~60 and 90% in old and new samples, respectively), confirming a previous study in sheep and goats (41). Notably, the prevalence of sheep with antibodies to BVDV strongly decreased between 2001 and 2016/2017, from 13.3 to 1.5%. This decrease is significant considering only the samples from canton of SZ (Supplementary Tables 5, 6), with 13.3 and 3.5% samples assigned to BVDV in the early and late sampling period, respectively. In return, the slight but significant increase in the prevalence ratio of BDV antibody-positive sheep in 2016/2017 compared to 2001 probably originates in the reduced level of BVDV-positive sheep rather than an increased risk of infection with BDV *per se*. Approximately half of the farms with BVDV antibody-positive sheep owned also animals with antibodies to BDV, whereas in the other half of these farms, the remaining animals were seronegative. As the time point of seroconversion in a given farm is unknown, purchase of seropositive sheep in the absence of circulating infections is a likely reason for this observation. This is exemplified by a farm sampled after the start of the eradication that had two animals with antibodies to BVDV and 31 seronegative sheep. These results provide strong evidence that BVD eradication in the Swiss cattle population led to a significant decrease in BVD seroprevalence in sheep, at least in Central Switzerland.

### Persistently Infected Cattle and Sheep Harbour Identical Subtypes of BDV

In the context of BVD eradication in cattle (15, 19), sequencing of the viruses from all BDV PI cattle revealed that they belong to only two BDV subgroups, i.e., 10 samples contained BDV-3 and 15 samples BDswiss. The latter subgroup was originally found exclusively in Switzerland distinct from any known BDV subgroup and, therefore, preliminarily termed “BDswiss” (8, 25). Subsequently, similar isolates were reported from Italy, and the subgroup was also named BDV-8 (45, 46). For the purpose of this study, we use both terms, i.e., BDswiss and BDV-8, to remain compatible to previous publications by us and by others. In phylogenetic analysis (Figure 2), the BDswiss (BDV-8) subgroup appears to be divided even in two distinct clades, hence sometimes labelled as BDswiss-A and BDswiss-B (22). However, separation into subgroups is just a useful tool, but does not represent an official terminology, as the current ICTV classification framework does not include a sub-genus category (47).

In sheep, only one single sample collected in 2017 in the canton of NW (Figure 1) was positive, and nucleotide sequencing showed that it clustered with samples from the sub-genotype BDswiss (BDV-8), one of the typical BD viruses found in Switzerland (Figure 2). As—inherently for this study—only one single time point from this animal was analysed, it cannot be concluded that this was a persistently infected animal. However, the lack of antibodies and the low Ct-value in real-time PCR (48) is indicative for this sheep being persistently infected. From this farm, additional 39 animals were sampled and tested for antibodies, yielding six negative or indeterminate and 33 positive results in ELISA. From the latter, all but one that was toxic in cell culture, could be assigned to BDV by cross-SNT, indicating that the virus-positive sheep might well have been the source of infection in this herd. The low prevalence of virus-positive sheep of <0.1% is in accordance with former studies that reported a virus prevalence in sheep of ~0.2 to 0.7% (44, 49–51). The shorter duration of pregnancy. i.e., resulting in a shorter time window to successfully induce a persistently infected lamb, and the lower life expectancy of persistently infected small ruminants (23) might be at the origin of the lower steady-state prevalence of PI sheep compared to cattle (20) in an endemic situation. Nevertheless, the low number of persistently infected sheep is sufficient to maintain the virus in the population due to the concomitant relatively low level of herd immunity, leaving a sufficient number of naïve, pregnant animals susceptible to infection.

Overall, a detailed sequence analysis was beyond the scope of this study, but analysis of the nucleotide sequence in the 5'-UTR provide strong evidence that in Switzerland, the same subgroups of BDV were found in cattle and sheep, and none of the other known BDV sub-genotypes (8) were ever detected in either cattle or sheep in Switzerland (unpublished observation). It is worth mentioning that the observations that we detected mainly just four BVDV-subgenotypes in Swiss cattle (21) and that the domestic pigs harbour a specific type of atypical porcine pestivirus (APPV) found exclusively in Switzerland to date (29), indicate that new types of pestiviruses were at least hitherto not successfully introduced into livestock in Switzerland (Figure 2). In cases where we identified a persistently infected sheep on a farm with a BDV PI calf (Table 2), the nucleotide sequence of the isolates from the cattle and the sheep were identical in the 5'-UTR. However, PI sheep were rarely found on the few farms investigated, which might be accounted to a time lag of several months between time point of virus transmission and investigation, leaving the identification of the source of infection often unresolved. Nevertheless, these observations argue against an independent circulation of BDV in cattle, in accordance with the contact to sheep being the highest risk factor for cattle to be positive to BDV or antibodies to BDV (22, 44). The transmission of pestivirus upon contact of cattle and sheep appear to occur rather infrequently, as we could not observe an increase in the number of BDV-infected cattle over time despite the strong decrease of pestivirus seroprevalence in cattle in recent years. Notably, only 3 out of 25 BDV PI cattle were detected in the years between 2013 and 2019. This drop in number might originate from the switch from antigen testing to antibody surveillance in 2013, and an intensification of the surveillance in 2018 due to an increase in the number of PI cattle detected in the previous year (15).

## Conclusions and Limitations

We identified only 24 cattle PI with BDV within ~10,000 samples analysed, which indicates that independent chains of infection, i.e., transmission of BDV from cattle to cattle, occurs only on rare occasions. In sheep, infections with pestiviruses remain endemic in the sheep population with BDV being the predominant viral antigen in sheep over all the years. However, the strong decrease in the prevalence of antibodies to BVDV in sheep in 2016/2017 compared to before the start of the BVDV control program indicates that BVD eradication in cattle is also of benefit for the sheep population. Thus, cross-species transmission of BVDV and BDV does occur between cattle and small ruminants and *vice versa* but only to a limited extent that does not appear to generally hamper the eradication of BVD in cattle on a national level. As data for both time points, i.e., before and after the start of BVD eradication in cattle, were only available for the Canton of Schwyz (SZ), we cannot, however, conclude that sheep in Switzerland in general profit from BVD eradication in cattle. Similarly, detailed information on cattle and sheep management in Swiss farms and the analysis of pestivirus prevalence in these premises would help to substantiate our conclusions, but such data are unfortunately not available. Nonetheless, as long as pestiviruses are not controlled in sheep, recurrent infections from sheep to cattle will continue to occur. Even if such transmission do not necessarily lead to the production of persistently infected animals, seroconversion upon transient infection will remain a hassle in the serological surveillance for the presence of BVDV in cattle. In case of detection of a calf persistently infected with BDV and, notably also BVDV, during the surveillance program, small ruminants need to be taken into account during epidemiological investigations to identify the source of infection as quickly as possible (15). It is therefore recommended to avoid repeated, close contact between sheep and cattle, which will not only prevent transmission of pestiviruses between the two species but will concomitantly reduce the occurrence of MCF in cattle.

## Data Availability Statement

The raw data supporting the conclusions of this article will be made available by the authors, without undue reservation.

## Ethics Statement

Ethical review and approval was not required for the animal study and written informed consent for participation was not obtained from the owners because blood samples were collected by national authorities in the frame of other control programs, and were just re-used for this study.

## Author Contributions

MS conceived and supervised the study and wrote the initial draft of the manuscript. AH and JS performed the experiments as part of their Master thesis with the support of JP and HS. CB and HS performed the sequencing and phylogenetic analysis. EdM performed the statistical analysis. All authors contributed to the analysis, interpretation of the results, reviewed the manuscript, and approved the final version.

## Funding

The project was financed by internal funds of the Institute of Virology and Immunology (IVI) with support of the Swiss Federal Food Safety and Veterinary Office (FSVO, project no. 1.17.04 to MS). EdM was supported by the Swiss National Science Foundation, grant no. 310030_172796 (to MS).

## Conflict of Interest

The authors declare that the research was conducted in the absence of any commercial or financial relationships that could be construed as a potential conflict of interest.

## Publisher's Note

All claims expressed in this article are solely those of the authors and do not necessarily represent those of their affiliated organizations, or those of the publisher, the editors and the reviewers. Any product that may be evaluated in this article, or claim that may be made by its manufacturer, is not guaranteed or endorsed by the publisher.
