# Supplementary material for: Benefit of Bovine Viral Diarrhoea (BVD) Eradication in Cattle on Pestivirus Seroprevalence in Sheep
Source: Front Vet Sci. 2021 Oct 4;8:681559. doi: 10.3389/fvets.2021.681559 (PMC8520948; doi:10.3389/fvets.2021.681559)
Supplement: Supplementary file 1 [file Data_Sheet_1.PDF]

Supplementary Material

Benefit of bovine viral diarrhoea (BVD) eradication in cattle on pestivirus seroprevalence in sheep

Andrea Franziska Huser[1#], Jessica Grace Schär[1#], Claudia Bachofen[3], Elena de Martin[1,2,4], Jasmine Portmann[1,2], Hanspeter Stalder[1,2], and Matthias Schweizer[1,2*]

[1] Institute of Virology and Immunology, Länggass-Str. 122, POB, CH-3001 Bern, Switzerland

[2] Department of Infectious Diseases and Pathobiology, Vetsuisse Faculty, University of Bern, CH-3012 Bern, Switzerland

[3] Institute of Virology, Vetsuisse Faculty, University of Zürich, Winterthurerstrasse 266a, CH-8057 Zürich, Switzerland

[4] Graduate School for Cellular and Biomedical Sciences, University of Bern, Switzerland.

[#] These authors contributed equally

Supplementary Table S1: Seroprevalence and number of farms affected per canton according to the *combined ELISA and SNT* results of sheep sera collected in 2001. Samples with inconclusive ELISA results were omitted from the calculation of the seroprevalence.

Canton	positive	negative	inconclusive	seroprevalence	Farms affected
SZ	228	962	31	19.2 %	72 (55 %)
UR	4	22	0	15.4 %	2 (100 %)
Total	232	984	31	19.1 %	74 (56 %)

Supplementary Table S2: Seroprevalence and number of farms affected per canton according to the *combined ELISA and SNT* results of sheep sera collected in 2016-2017. Samples with inconclusive ELISA results were omitted from the calculation of the seroprevalence.

Canton	positive	negative	inconclusive	seroprevalence	Farms affected
LU	5	277	5	1.8 %	1 (6 %)
NW	41	61	1	40.2 %	4 (100 %)
OW	39	83	1	32.0 %	3 (33 %)
SZ	81	509	27	13.7 %	14 (48 %)
UR	60	287	14	17.3 %	9 (50 %)
ZG	35	58	0	37.6 %	2 (40 %)
Total	261	1275	48	17.0 %	33 (40 %)

Supplementary Table S3: Interpretation and summary of the cross-SNT results of the sera samples in 2001 (related to Table 5).

Evaluation BVDV-1a & BDV	Evaluation BVDV-1h & BDV	Assignment	n	Proportion per assignment [%]	Proportion overall [%]
BVDV	BVDV		4	14.3	
indeterminate	BVDV	BVDV	18	64.3	13.3
negative	BVDV		6	21.4	
BDV	BDV		88	68.6	
BDV	indeterminate	BDV	39	30.5	60.7
indeterminate	BDV		1	0.8	
BDV	BVDV		1	5.0	
indeterminate	indeterminate	indeterminate	16	80.0	9.5
indeterminate	negative		1	5.0	
negative	indeterminate		2	10.0	
negative	negative	negative	35	100	16.6
Total assignable			211		100
Toxic sera			54		
unavailable			2		
Total sera positive in ELISA			267		

Supplementary Table S4: Interpretation and summary of the cross-SNT results of the sera samples in 2016/2017 (related to Table 5).

Evaluation BVDV-1a & BDV	Evaluation BVDV-1h & BDV e	Assignment	n	Proportion per assignment [%]	Proportion overall [%]
indeterminate	BVDV	BVDV	3	75.0	1.5
negative	BVDV		1	25.0	
BDV	BDV		189	78.1	
BDV	indeterminate	BDV	52	21.5	90.0
indeterminate	BDV		1	0.4	
BVDV	BDV	indeterminate	1	50.0	0.7
indeterminate	indeterminate		1	50.0	
negative	negative	negative	21	100	7.8
Total assignable			269		100
Toxic sera			13		
Total sera positive in ELISA			282		

Supplementary Table S5: Interpretation and summary of the cross-SNT results of the sera samples in 2001 in the canton of Schwyz (SZ) (related to Supplementary Table 3).

**Evaluation BVDV-1a & BDV**	**Evaluation BVDV-1h & BDV**	**Assignment**	n	**Proportion per assignment [%]**	**Proportion overall [%]**
BVDV	BVDV	BVDV	4	14.3	13.3
indeterminate	BVDV		18	64.3	
negative	BVDV		6	21.4	
BDV	BDV	BDV	88	68.6	60.7
BDV	indeterminate		37	30.5	
indeterminate	BDV		1	0.8	
BDV	BVDV	indeterminate	1	5.0	9.5
indeterminate	indeterminate		15	80.0	
indeterminate	negative		1	5.0	
negative	indeterminate		2	10.0	
negative	negative	negative	34	100	16.6
Total assignable			207		100
Toxic sera			53		
unavailable			2		
Total sera positive in ELISA			262		

Supplementary Table S6: Interpretation and summary of the cross-SNT results of the sera samples in 20016/2017 in the canton of Schwyz (SZ) (related to Supplementary Table 4).

Evaluation BVDV-1a & BDV	Evaluation BVDV-1h & BDV e	Assignment	n	Proportion per assignment [%]	Proportion overall [%]
indeterminate	BVDV	BVDV	3	100.0	3.5
negative	BVDV		0	0.0	
BDV	BDV	BDV	63	81.8	89.5
BDV	indeterminate		13	16.9	
indeterminate	BDV		1	1.3	
BVDV	BDV	indeterminate	1	100.0	1.2
indeterminate	indeterminate		0	0.0	
negative	negative	negative	5	100	5.8
Total assignable			86		100
Toxic sera			0		
Total sera positive in ELISA			86		

Supplementary Table S7: All sequences of the 5'-UTR regions were obtained from GenBank or were determined in this study. The alignment was performed using the MAFFT online service with the default settings (1). The evolutionary history was inferred using the Neighbour-Joining method (2) with bootstrap values based on 1000 replicates (3). Only bootstrap values ≥ 99 are shown in the phylogenetic tree. The tree is drawn to scale, with branch lengths in the same units as those of the evolutionary distances used to infer the phylogenetic tree. The evolutionary distances were computed using the number of differences method (4) and are in the units of the number of base differences per sequence. The analysis involved 48 nucleotide sequences. All ambiguous positions were removed for each sequence pair. There were a total of 277 positions in the final dataset. Evolutionary analyses were conducted in MEGA7 (5).

Genotype	Canton	Year of isolation	Accession no.	Isolate
APPV	BE	2015	MN099167	5620
APPV	LU	2006	MN099163	8247
APPV	LU	2018	MN099170	180416
BDV3	GR	2015	MH908092	boBD-CH15
BDV3	TG	2016	MH908093	boBD-CH16
BDV3	UR	2019	MW659877	boBD-CH19
BDV3	GR	2009	MH908079	boBD-CH2
BDV3	UR	2019	MW659878	boBD-CH20
BDV3	GR	2020	MW659879	boBD-CH21
BDV3	TG	2020	MW659881	boBD-CH23
BDV3	TG	2020	MW659882	boBD-CH24
BDV3	UR	2010	MH908080	boBD-CH3
BDV3	SZ	2008	MH908082	boBD-CH5
BDVswiss-A	SG	2009	MH908078	boBD-CH1
BDVswiss-A	SZ	2012	MH908085	boBD-CH10
BDVswiss-A	SZ	2012	MH908086	boBD-CH11a
BDVswiss-A	SZ	2012	MH908087	boBD-CH11b
BDVswiss-A	LU	2012	MH908089	boBD-CH13a
BDVswiss-A	LU	2013	MH908090	boBD-CH13b
BDVswiss-A	ZG	2015	MH908091	boBD-CH14
BDVswiss-A	BE	2019	MW659875	boBD-CH17
BDVswiss-A	LU	2019	MW659876	boBD-CH18
BDVswiss-A	SG	2020	MW659880	boBD-CH22
BDVswiss-A	SZ	2010	MH908081	boBD-CH4
BDVswiss-A	ZG	2011	MH908083	boBD-CH8
BDVswiss-A	GR	2011	MH908084	boBD-CH9
BDVswiss-A	NW	2017	MW659888	mm1455
BDVswiss-A	SZ	2011	MF102261	R9336/11
BDVswiss-B	SZ	2012	MH908088	boBD-CH12
BVDV1b	AG	2008	MH900629	CH0007

BVDV1b	GR	2008	MH900969	CH0347
BVDV1b	SZ	2009	MH901152	CH0530
BVDV1b	VD	2011	MH901246	CH0624
BVDV1e	AG	2008	MH901291	CH0669
BVDV1e	AG	2008	MH901343	CH0721
BVDV1e	BE	2008	MH901666	CH1044
BVDV1e	BL	2008	MH902141	CH1519
BVDV1h	AG	2008	MH903564	CH2942
BVDV1h	AI	2009	MH903737	CH3115
BVDV1h	BE	2009	MH904320	CH3698
BVDV1h	GR	2008	MH904824	CH4202
BVDV1k	AG	2008	MH907215	CH6593
BVDV1k	AG	2008	MH907225	CH6603
BVDV1k	FR	2010	MH907493	CH6871
BVDV1k	SO	2008	MH907830	CH7208
CSFV	ns	1993	AF045068	1/93
CSFV	ns	1993	AF045069	2/93
CSFV	ns	1993	AF045070	3/93/1

ns: not specified.

References:

1. Katoh K, Rozewicki J, Yamada KD. MAFFT online service: multiple sequence alignment, interactive sequence choice and visualization. *Brief Bioinform* (2019) 20:1160-6. doi: 10.1093/bib/bbx108
2. Saitou N, Nei M. The neighbor-joining method: a new method for reconstructing phylogenetic trees. *Mol Biol Evol* (1987) 4:406-25. doi: 10.1093/oxfordjournals.molbev.a040454
3. Felsenstein J. Confidence limits on phylogenies: an approach unsing the bootstrap. *Evolution* (1985) 39:783-91. doi: 10.1111/j.1558-5646.1985.tb00420.x
4. Nei M, Kumar S. *Molecular evolution and phylogenetics*. New York: Oxford University Press (2000).
5. Kumar S, Stecher G, Tamura K. MEGA7: Molecular evolutionary genetics analysis version 7.0 for bigger datasets. *Mol Biol Evol* (2016) 33:1870-4. doi: 10.1093/molbev/msw054
